# Impaired Cardiorespiratory Fitness in Cancer Survivors: Time to Come Together to Solve a Complex Problem

**DOI:** 10.1016/j.jaccao.2025.01.002

**Published:** 2025-02-18

**Authors:** Kate A. Bolam, Erin J. Howden

**Affiliations:** Cardiometabolic and Exercise Physiology Lab, Baker Heart and Diabetes Institute, Melbourne, Victoria, Australia

**Keywords:** alkylating therapy, anthracycline, a-Vo_2_ difference, cancer survivorship, cardiopulmonary fitness, exercise, treatment, Vo_2_peak

**Figure:**
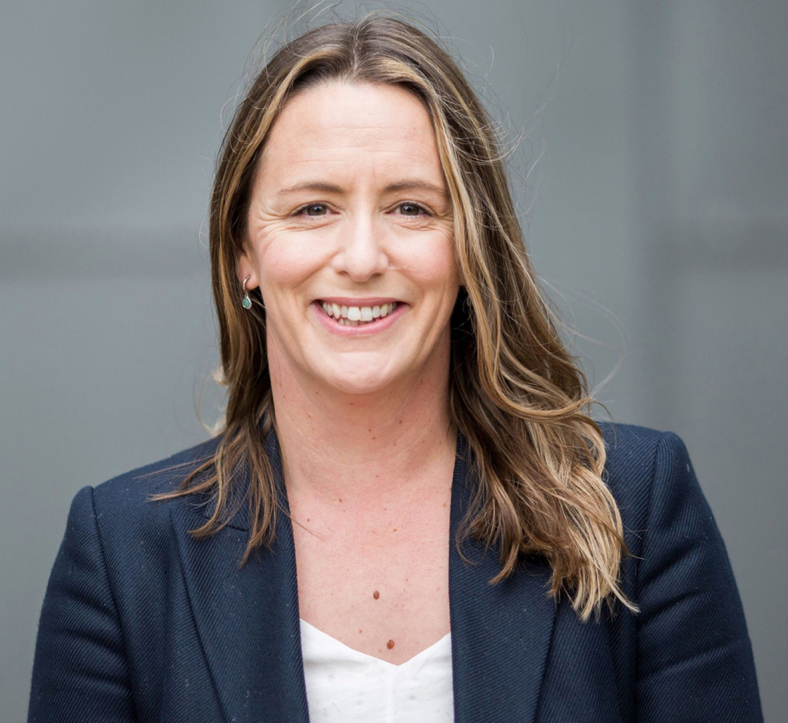

Globally, the number of cancer survivors is increasing, with 70% of people diagnosed with cancer living more than 5 years from diagnosis. These outstanding oncological outcomes have consequently led to a widened focus for survivors, health care professionals, researchers, and health care systems to better identify and provide supportive care to manage the growing burden of complex health needs of cancer survivors. It is well accepted that anthracycline-containing chemotherapy can lead to cardiotoxicity and ultimately heart failure, but increasingly we are seeing other chronic health conditions normally associated with older aging manifesting earlier in cancer survivors. Combined with the aging population, the growing global population of cancer survivors, who are also generally living longer, presents an urgent public health challenge that requires increased focus.

Significant attention has been focused on the cardiac complications of certain anticancer therapies, but doing so fails to acknowledge that many therapies can affect multiple systems. Cardiorespiratory fitness, which can be measured objectively as peak oxygen uptake (Vo_2_peak), is reflective of the integrated physiology of several systems from the cardiopulmonary system to the cellular level and affects an individual’s capacity to perform activities of daily living.^1^ An individual’s oxygen uptake (Vo_2_) for a given activity reflects a highly coordinated system that involves transporting oxygen from the atmosphere to the mitochondria within the skeletal muscle, where the production of energy in addition to other enzymatic reactions that require oxygen takes place.^2^ Thus, objectively measured Vo_2_peak can provide mechanistic insights into the systemic effects of anticancer therapy. Additionally, Vo_2_peak is a powerful predictor of future disease burden and mortality.^1^ Thus, this measure has the potential to provide novel insights into future chronic disease burden beyond cardiac focused modalities.

In this issue of *JACC: CardioOncology*, Johansen et al^3^ report the findings from their systematic review and meta-analysis to evaluate the effects of systemic cancer therapies on Vo_2_peak across the cancer survivorship continuum. The study was preregistered with the International Prospective Register of Systematic Reviews and conducted in accordance with the Preferred Reporting Items for Systematic Reviews and Meta-Analyses guidelines. To address their aims, the investigators included randomized and nonrandomized trial designs focusing on the nonintervention control groups of such studies, prospective cohort studies with pre- and post-treatment assessments, and cross-sectional studies with post-treatment assessments that included a noncancer control group. Additional inclusion criteria were adults with adult-onset cancer, regardless of stage, and adult survivors of any (childhood) cancer who received systemic anticancer treatment) and a direct measurement of Vo_2_peak before and/or after systemic therapy. The search returned 5,126 records, of which 44 met the inclusion criteria and were included in the analysis. Of the 44 studies included, there were 27 prospective and 17 cross-sectional studies. Just under one-half of the studies (48%) focused solely on survivors of breast cancer, with chemotherapy as the treatment modality in the majority (90%) of studies. Across the included studies, there were 2,606 cancer survivors (median age 52.7 years) and 1,923 noncancer control participants (median age 56.0 years) from studies of relatively small sample sizes.

The key findings are that cancer survivors across all stages of the survivorship trajectory experienced reduced Vo_2_peak following systemic treatment. Short-term systemic therapy over an approximately 13-week period acutely lowered Vo_2_peak by about 10% (weighted mean difference −2.13 mL · kg^−1^ · min^−1^; 95% CI: −2.76 to −1.50 mL · kg^−1^ · min^−1^; *I*^2^ = 48%). Studies of longer term follow-up (range: 6 weeks to 12 years) after systemic therapy showed that Vo_2_peak was markedly lower than in noncancer control subjects (weighted mean difference −6.39 mL · kg^−1^ · min^−1^; 95% CI: −7.60 to −5.18 mL · kg^−1^ · min^−1^; *I*^2^ = 61%). Interestingly, the difference in Vo_2_peak between those with and without cancer in the short term (∼14.6 months post-treatment) compared with the long term (∼8.4 years) did not differ significantly, indicating that the greatest declines are occurring earlier post-treatment, and there is lack of recovery in the long term. This study attempted to differentiate the response of Vo_2_peak in different cancer types following treatment. The available prospective evidence was limited to upper gastrointestinal, breast, and colorectal cancers, and when compared with pretreatment values, Vo_2_peak was reduced significantly across all cancer types. Interestingly, although there was no statistically significant difference among cancer types, there was a tendency toward a greater reduction in Vo_2_peak among breast cancer survivors (*P* = 0.059). Ultimately it would be informative to understand the effects of all systemic therapies on Vo_2_peak, but most patients during treatment will receive a combination of anticancer therapies and supportive therapies and have any number of patient-related factors that could influence their responses. Indeed, evaluating the impact of treatment type was a prespecified subgroup analysis, but insufficient reporting and small sizes prevented this analysis.

As the investigators state, it is vital that we gain a more comprehensive understanding of the cascade of physiological changes associated with systemic therapies that contribute to the decrease in Vo_2_peak if we hope to develop effective counter therapies. Mechanisms underpinning reduced Vo_2_peak were assessed in 4 studies, and the analysis identified that lower Vo_2_peak was associated with lower arterial venous oxygen (a-Vo_2_) difference but not peak cardiac output, suggesting that exercise intolerance in cancer survivors may be partially explained by impairment in peripheral function (ie, oxygen diffusion) rather than a centrally mediated impairment. However, caution should be applied when using correlative analysis to interpret mechanisms of impairment, given that the variables of interest are in no way independent. For example, according to the Fick equation, Vo_2_ is equal to the product of cardiac output and a-Vo_2_ difference. Thus, a-Vo_2_ difference can be derived from the ratio of cardiac output to Vo_2_. This approach can be used to gain insight into the contributors to reduced Vo_2_peak, but further work is required using more direct measurements of skeletal muscle quality and function (and other peripheral components), preferably during exercise and at multiple time points throughout survivorship to determine the role of the periphery in impairing cardiorespiratory fitness.

## Future Areas of Development

Although exercise training holds promise as an effective adjunctive therapy to prevent declines and improve Vo_2_peak in cancer survivors,^4^ the most effective dose and timing of exercise are unclear (eg, before, during, or after systemic therapy). Likely because of the considerable costs and complicated logistics of conducting larger and longer term (>5- to 10-year follow-up) randomized controlled exercise trials, current exercise oncology guidelines rely on findings from smaller and shorter exercise interventions often developed to counter a multitude of side effects rather than cardiorespiratory fitness specifically. Alternatively, the findings in this systematic review show that it would be prudent to trial supportive care strategies, such as exercise and/or exercise plus pharmacotherapies, for cancer survivors undergoing systemic therapies at treatment initiation and well into survivorship to counter the decreases in Vo_2_peak. Additionally, individuals with or at risk for reduced Vo_2_peak may benefit from more targeted exercise prescriptions and at greater doses than recommended in current guidelines.^5^ Larger, comprehensive national and/or international studies evaluating potential interventions such as exercise or combined exercise with pharmacotherapy and other lifestyle interventions are essential to inform future survivorship guidelines.

It is vital that future trials use sophisticated methodologies (eg, stress echocardiography, exercise cardiac magnetic resonance imaging) to investigate whether central or peripheral mechanisms are the predominant contributors to this issue. Indeed, this integrative approach has revealed novel insights into the mechanisms of reduced cardiorespiratory fitness in other complex conditions.^6^ Differentiating the mechanism of cardiorespiratory impairment will enable the development of more effective precision-based countermeasures for this clinical subgroup.

Although it was not the focus of this systematic review, given the ever increasing challenges placed upon health systems globally, future research in this field must work toward identifying who is most at risk for experiencing this decline in Vo_2_peak. Doing so will enable the channeling of health care resources to those who need them the most in the hope of preventing serious and long-term health complications.

## Funding Support and Author Disclosures

Dr Howden has consulted for Race Oncology. Dr Bolam has reported that she has no relationships relevant to the contents of this paper to disclose.

## References

[1] Ross R., Blair S.N., Arena R. (2016). Importance of assessing cardiorespiratory fitness in clinical practice: a case for fitness as a clinical vital sign: a scientific statement from the American Heart Association. Circulation.

[2] Dillon H.T., Foulkes S.J., Baik A.H. (2024). Cancer therapy and exercise intolerance: the heart is but a part: *JACC: CardioOncology* state-of-the-art review. JACC CardioOncol.

[3] Johansen S.H., Wisløff T., Edvardsen E. (2025). Effects of systemic anticancer treatment on cardiorespiratory fitness: a systematic review and meta-analysis. JACC CardioOncol.

[4] Scott J.M., Zabor E.C., Schwitzer E. (2018). Efficacy of exercise therapy on cardiorespiratory fitness in patients with cancer: a systematic review and meta-analysis. J Clin Oncol.

[5] Campbell K.L., Winters-Stone K.M., Wiskemann J. (2019). Exercise guidelines for cancer survivors: consensus statement from international multidisciplinary roundtable. Med Sci Sports Exerc.

[6] Howden E.J., Ruiz-Carmona S., Claeys M. (2021). Oxygen pathway limitations in patients with chronic thromboembolic pulmonary hypertension. Circulation.

